# Local Properties of Vigilance States: EMD Analysis of EEG Signals during Sleep-Waking States of Freely Moving Rats

**DOI:** 10.1371/journal.pone.0078174

**Published:** 2013-10-22

**Authors:** Rupesh Kumar, Ram Ramaswamy, Birendra Nath Mallick

**Affiliations:** 1 School of Computational and Integrative Sciences, Jawaharlal Nehru University, New Delhi, India; 2 School of Life Sciences, Jawaharlal Nehru University, New Delhi, India; 3 University of Hyderabad, Central University P.O., Hyderabad, India; Universidade do Estado do Rio de Janeiro, Brazil

## Abstract

Understanding the inherent dynamics of the EEG associated to sleep-waking can provide insights into its basic neural regulation. By characterizing the local properties of the EEG using power spectrum, empirical mode decomposition (EMD) and Hilbert-spectral analysis, we can examine the dynamics over a range of time-scales. We analyzed rat EEG during wake, NREMS and REMS using these methods. The average instantaneous phase, power spectral density (PSD) of intrinsic mode functions (IMFs) and the energy content in various frequency bands show characteristic changes in each of the vigilance states. The 2nd and 7th IMFs show changes in PSD for wake and REMS, suggesting that those modes may carry wake- and REMS-associated cognitive, conscious and behavior-specific information of an individual even though the EEG may appear similar. The energy content in θ_2_ (6Hz-9Hz) band of the 1st IMF for REMS is larger than that of wake. The decrease in the phase function of IMFs from wake to REMS to NREMS indicates decrease of the mean frequency in these states, respectively. The rate of information processing in waking state is more in the time scale described by the first three IMFs than in REMS state. However, for IMF5-IMF7, the rate is more for REMS than that for wake. We obtained Hilbert-Huang spectral entropy, which is a suitable measure of information processing in each of these state-specific EEG. It is possible to evaluate the complex dynamics of the EEG in each of the vigilance states by applying measures based on EMD and Hilbert-transform. Our results suggest that the EMD based nonlinear measures of the EEG can provide useful estimates of the information possessed by various oscillations associated with the vigilance states. Further, the EMD-based spectral measures may have implications in understanding anatamo-physiological correlates of sleep-waking behavior and clinical diagnosis of sleep-pathology.

## Introduction

Since its discovery by Berger [1], the electroencephalogram (EEG) has been recognized as an important tool in psycho-behavioral studies and sleep research. It is the commonest and the most characteristic feature for objectively defining various stages of sleep-waking. Cortical activation is shown by EEG desynchronization, while sleep-spindles and slow waves (0.4Hz-4Hz) are the hallmark of EEG during behavioral arousal (wakefulness) and non-rapid eye movement sleep (NREMS) respectively. The rapid eye movement sleep (REMS) is characterized by EEG desynchronization, apparently similar to that shown in wakefulness, atonia of anti-gravity muscles, and rapid eye movements observed in the electrooculogram (EOG).

During NREMS the neuronal firing patterns change from rapid firing, characteristic of arousal, to low frequency synchronized rhythms [2]. It was believed that these rhythms are generated due to reciprocal interactions in thalamo-cortical neural networks [2,3]. The ascending reticular activating system, comprising of neurons and their connections in the brainstem core, is necessary for the tonic maintenance of cortical activation [4] when awake. Early stages of sleep are characterized by the presence of α-waves (7Hz-14Hz) in the EEG, which changes to slow oscillations (0.1Hz-4Hz) as sleep deepens. REMS is characterized by abolition of low-frequency oscillations and an increase in cellular excitability apparently comparable to that as in wakefulness [5]. Central neurotransmission plays a key role in the modulation of the EEG. For example, cholinergic and noradrenergic neurotransmission can affect the EEG slow wave activity by blocking the postsynaptic receptors [6,7]. Disruption of any of the neurotransmission systems can lead to alteration of EEG as seen in pathological or cognitive disorders [8–11]. Therefore, analyzing the dynamical properties of the EEG can provide insights into understanding the mechanism of its basic regulation [12].

Traditional methods such as the fast Fourier transform (FFT) and wavelet analysis have been extensively employed to study various features in the EEG during sleep [13–16]. Parameters that can be calculated through these methods have been shown to have implications in assessing patho-physiological states. For instance EEG slow wave activity (SWA), namely spectral power in the frequency range 0.4Hz-4HZ is an important parameter to estimate sleep homeostasis and is a function of the duration of prior wakefulness [17]. Borbély proposed a two process model of sleep wake regulation that is based on SWA and the circadian rhythm. This model [18] postulates that the architecture and propensity of sleep are determined by the interaction of two constituent processes: the homeostatic process S which correlates with SWA and the circadian process C which correlates with sleep timing. The process S increases as an exponentially saturating function during waking and it decays exponentially during sleep. The upper (sleep onset) and lower (sleep termination) thresholds of the process S are controlled by the process C which, being circadian, is independent of sleep and waking [18,19]. Experiments as well as simulation studies of this model explain diverse phenomena such as rebound sleep after sleep deprivation, internal desynchronization in the absence of time cues, sleep fragmentation under continuous bed rest, sleep during shift work and circadian phase dependence of sleep duration [20–22]. Spectral power and high-voltage spindle analysis could serve as a useful tool for evaluating the efficacy of pharmacological strategies aimed at alleviating the neurotransmission deficit [23]. 

However, traditional methods are limited by both fundamental as well as pragmatic considerations, and are relatively worse for discriminating the changes in wake and REMS states [24] due to the nonlinear and non-stationary structure of EEG signals. It should be pointed out that several nonlinear dynamical tools have been employed earlier in the analysis of EEG during sleep-wake stages [25–28]. Measures based on nonlinear dynamics have not been uniformly successful in providing a faithful classification protocol for the wake and REMS states, and therefore, failed to characterize the dynamics or mechanism that regulates these states. The present study is an attempt to understand the dynamical aspects of the EEG. Adaptive methods such as empirical mode decomposition (EMD) have been applied to rat EEG recordings associated to various vigilance states. EMD is a fully data driven method that acts locally in time. A given signal is analyzed in terms of its intrinsic modes of oscillation: these are the intrinsic mode functions (IMFs). The IMFs were then further analyzed to obtain quantitative measures such as power spectral density (PSD), average Hilbert phase, and Hilbert-Huang spectral entropy (HHSE). 

## Materials and Methods

### Surgical details and EEG recording

Studies were conducted strictly following the National Institutes of Health guidelines for the care and use of laboratory animals and the experiments were approved by the Institutional Animal Ethics Committee of Jawaharlal Nehru University, India. All experiments were conducted on chronically implanted freely moving male Wistar rats (n=12) of weight 280g-320g. Rats were maintained at 12:12 light: dark cycle (lights on at 7:00 AM) and ambient temperature 24±10 °C with food and water *ab libitum*. The rats were surgically prepared for chronic sleep-wake recording as reported earlier [29]. In brief, under surgical anesthesia (ketamine hydrochloride 80 mg/kg and xylazine hydrochloride 10 mg/kg i.p.), four stainless steel screw electrodes were fixed on the skull for recording bilateral EEG. Two screw electrodes were placed at 2.0 mm rostral and 2.0 mm lateral (frontal bone), while two others were fixed at 2.0 mm caudal and 2.0 mm lateral (parietal bone) to the bregma. Another screw electrode was implanted over the frontal sinus to serve as the animal ground. Electrodes (flexible wires insulated except at the tip) were connected bilaterally to dorsal neck muscles and muscles near the external canthus of the eyes to record bilateral electromyogram (EMG) and EOG respectively. Leads from all the electrodes were soldered to a nine-pin connector and the complete assembly was anchored to the skull with dental acrylic.

Rats were allowed to recover from surgery for at least one week. During the recovery days the rats were adapted to the recording chamber and cables. On the day of recording an animal was placed in a semi sound-proof Faraday cage and was connected to a recording cable that was lightly suspended above them by a counter-weighted beam. The recording was done for 5-6 h (beginning at 9:00 AM) each day. During experiments, EEG, EMG and EOG signals were recorded continuously using a polysomnographic recording device (Embla; Medcare Flaga Medical Devices, Reykjavik, Iceland). The electrophysiological signals were digitally sampled at a frequency of 100Hz and stored in a computer using the Embla recording device (Somnologica Studio; Medcare Flaga Medical Devices).

### Sleep Scoring

**Figure 1:**
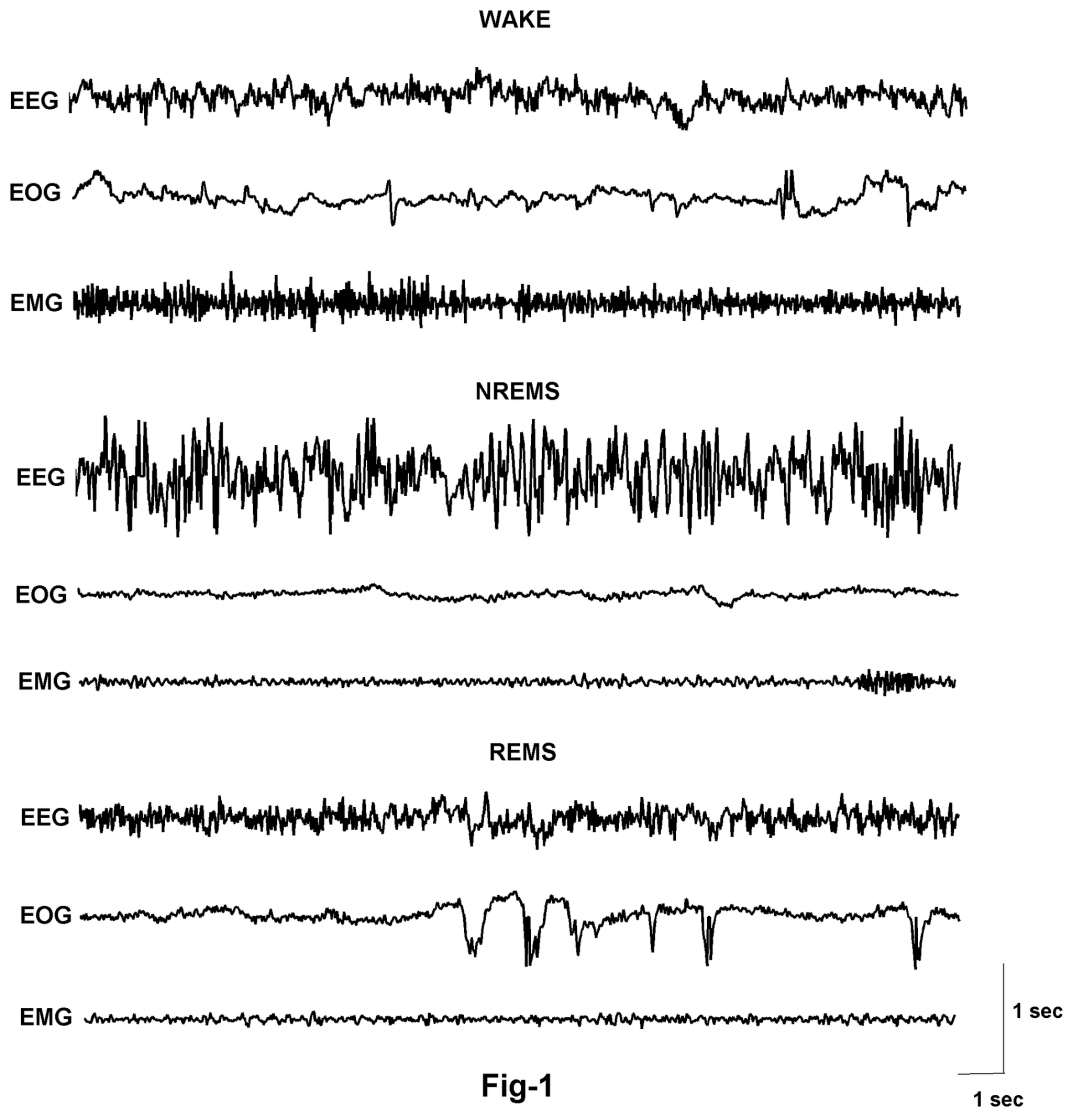
Typical single channel fronto-frontal EEG signals during different vigilance states (Wake, NREMS, and REMS). Standard acronyms are used, EEG: Electroencephalogram, EOG: Electrooculogram, EMG: Electromyogram.

Vigilance states were manually scored offline using 10s epochs and were subdivided into active wake, NREMS and REMS as reported earlier [29,30]. The waking state was identified by the presence of desynchronized EEG accompanied by a high EMG tone and/or muscle movement and eye movements in the EOG. NREMS was characterized by EEG synchronization (>75% epoch) and the appearance of spindle in EEG, no active muscle movement in EMG and reduced eye movements in EOG. REMS was identified using EEG desynchronization, muscle atonia and frequent eye movements, usually following NREMS (Figure 1). Based on the physiological sleep profile the representative collections of 10 sec epochs (n = 240 for each stage) of the wake, NREMS and REMS stages were subjected to programs written in MATLAB for further analysis.

### EEG analysis

#### Power Spectral Analysis

From the Fourier transform of the EEG signal,*F*(*ω*) , the PSD *S*(*ω*)was calculated for the identified 10s EEG epochs in different sleep-wake states (described above). The mean PSD profile for each stage is given by

$$S(\omega )=\langle F(\omega )F^{*}(\omega )\rangle$$ (1)

Where, 〈 .〉 denotes an average over the individual epochs. In each of the states, only those scoring epochs that were free from artifacts were included. As mentioned earlier, the NREMS scoring epochs were chosen based on high spindle density by visual inspection (Figure 1).

#### Empirical Mode Decomposition and Hilbert transform

The EMD analyses a given signal *s*(*t*) into a series of component waveforms termed the IMF [31]. Each IMF, obtained by a standard *sifting process*, is characterized as having a number of extrema and number of zero-crossings that was identical or differs by one. Secondly the mean value of the envelope defined by local maxima and envelope defined by local minima is zero. Each IMF thus obtained satisfies completeness and orthogonality and represents the modulation of a certain frequency at a specific time scale. In this study, we used an improved EMD code as described by Rilling and Goncalves [32]. The EMD algorithm is described as follows

i) Identify the extrema of the signal*s*(*t*).
ii) Connect all the maxima by a cubic spline interpolation to find the upper envelope*e*_*max*_(*t*). Similarly, find the lower envelope *e*_*min*_(*t*) by using minima.
iii) Calculate the mean envelope$m(t)=\frac{e_{\max}(t)+e_{\min}(t)}{2}$.
iv) Subtract this mean envelope from the signal to obtain the detail*h*(*t*)=*S*(*t*)-*m*(*t*).
v) This detail *h*(*t*) is checked for IMF characteristics mentioned above. For robust and efficient calculation of IMF, a stopping criteria is used [31,32]. If this criterion is satisfied, the first IMF is given by*C*_1_=*h*(*t*), else the residual *h*(*t*) is treated as the original signal*s*(*t*), and one returns to step (ii). The process described in (i)-(v) is called *sifting*.

The stopping criterion for sifting [32] is based on setting a threshold to the evolution function *σ*(*t*)=|*m*(*t*)/*a*(*t*)| where*a*(*t*)=(*e*_*max*_(*t*)−*e*_*min*_(*t*))/2. The usual strategy to take as *σ*(*t*)<*θ*_1_ for some prescribed fraction 1−*α*of the total duration, while *σ*(*t*)<*θ*_2_for the remaining. The values*α*=0.05,*θ*_1_=0.05 , *θ*_2_=0.5are used here.

vi) Compute the residual signal *r*_1_(*t*)=*s*(*t*)-*C*_1_(*t*) and subject to subsequent IMFs(*C*_*i*_(*t*)), treating *r*_*i*_(*t*)as the original signal using the above procedure (i)-(v) . The decomposition terminates when the residual becomes monotonic and from which no further IMFs can be extracted.

The signal *s*(*t*) can thus be represented as a sum of *n* IMFs, *C*_*i*_(*t*)and a residual *r*(*t*)

$$s(t)=\sum_{i=1}^{N}C_{t}(t)+r_{}(t)$$ (2)

The Hilbert–Huang transform is a two–step process, the first being the above decomposition of the signal into its intrinsic mode functions using EMD. The second step is to construct the Hilbert spectrum of each IMF by applying the Hilbert transform; this provides an energy frequency-time distribution. The analytical signal corresponding to *s*(*t*) _is given by [33]_ .

$$\psi (t)=s(t)+i\tilde{s}(t)=A(t)e^{i\varphi (t)}$$ (3)

Where, $\tilde{s}(t)$is the Hilbert transform of *s*(*t*), namely

$$\tilde{s}(t)=\frac{1}{\pi }P.V.\int_{-\infty }^{+\infty }\frac{s(t^{\prime })}{t-t^{\prime }}dt^{\prime }$$ (4)

*P.V.* denotes the Cauchy principal value. The quantities

$$A(t)={\sqrt{s{\left(t\right)}^{2}+\tilde{s}{\left(t\right)}^{2}}}^{}$$ (5)

and

$$\varphi (t)=\tan^{-1}\left(\frac{\tilde{s}(t)}{s(t)}\right)$$ (6)

define the amplitude and phase of the analytic signal. When *s*(*t*) is aperiodic (as is typically the case for IMFs) the frequency $\omega (t)=\dot{\varphi }(t)$ is not constant. Indeed, the nature of its variation reveals much about the dynamics [34]. Together with the instantaneous frequency *ω*(*t*) and Eqs. (2,3), we obtain the Hilbert spectrum *H*(*ω*,*t*) that gives the likelihood of the exact occurrence time of a specific frequency of oscillation. The total amplitude contribution from each frequency value can be calculated from the marginal Hilbert spectrum given by

$$h(\omega )=\int_{0}^{T}H(\omega ,t)dt$$ (7)

Where, T is the total time duration of the time series. We note that applying the Hilbert transform to IMFs is meaningful since they satisfy the condition of being symmetric with respect to the local zero mean. The instantaneous phase of the oscillations can be found from the analytic signal thus constructed.

Spectral entropy measures have recently been used to monitor the depth of anesthesia in humans [35]. We used the entropy measures from Hilbert transform to evaluate the information processing during vigilance states. From the normalized marginal Hilbert transform, namely

$$\hat{h}(\omega )=\frac{h(\omega )}{\sum_{\omega }h(\omega )}$$ (8)

it is possible to obtain the Hilbert-Huang spectral entropy (HHSE), denoted *S*_*H*_as

$$S_{H}=\frac{-\sum_{\omega }\hat{h}(\omega )\log(\hat{h}(\omega ))}{\log m}$$ (9)

Where, *m* is the number of frequency components.

We calculated the power spectral densities, relative power in various frequency bands, δ (0.4Hz-4Hz), θ_1_ (4Hz-6Hz), θ_2_ (6Hz-9Hz), α (9Hz-14Hz), β_1_ (14Hz-20Hz) and β_2_ (20Hz-45Hz) and the instantaneous phase of the IMFs for further analysis. 

### Statistical Analysis

Statistical analysis (of the results section below) was done as follows: the total power in each of the frequency bands δ, θ_1_, θ_2_, α, β_1_ and β_2_ in each of the vigilance states was averaged over all epochs to obtain the mean and standard deviation. One-way ANOVA was performed with the vigilance states as a factor on the mean PSD of each frequency bands and HHSE values. 

## Results

### EEG Power spectrum

**Figure 2:**
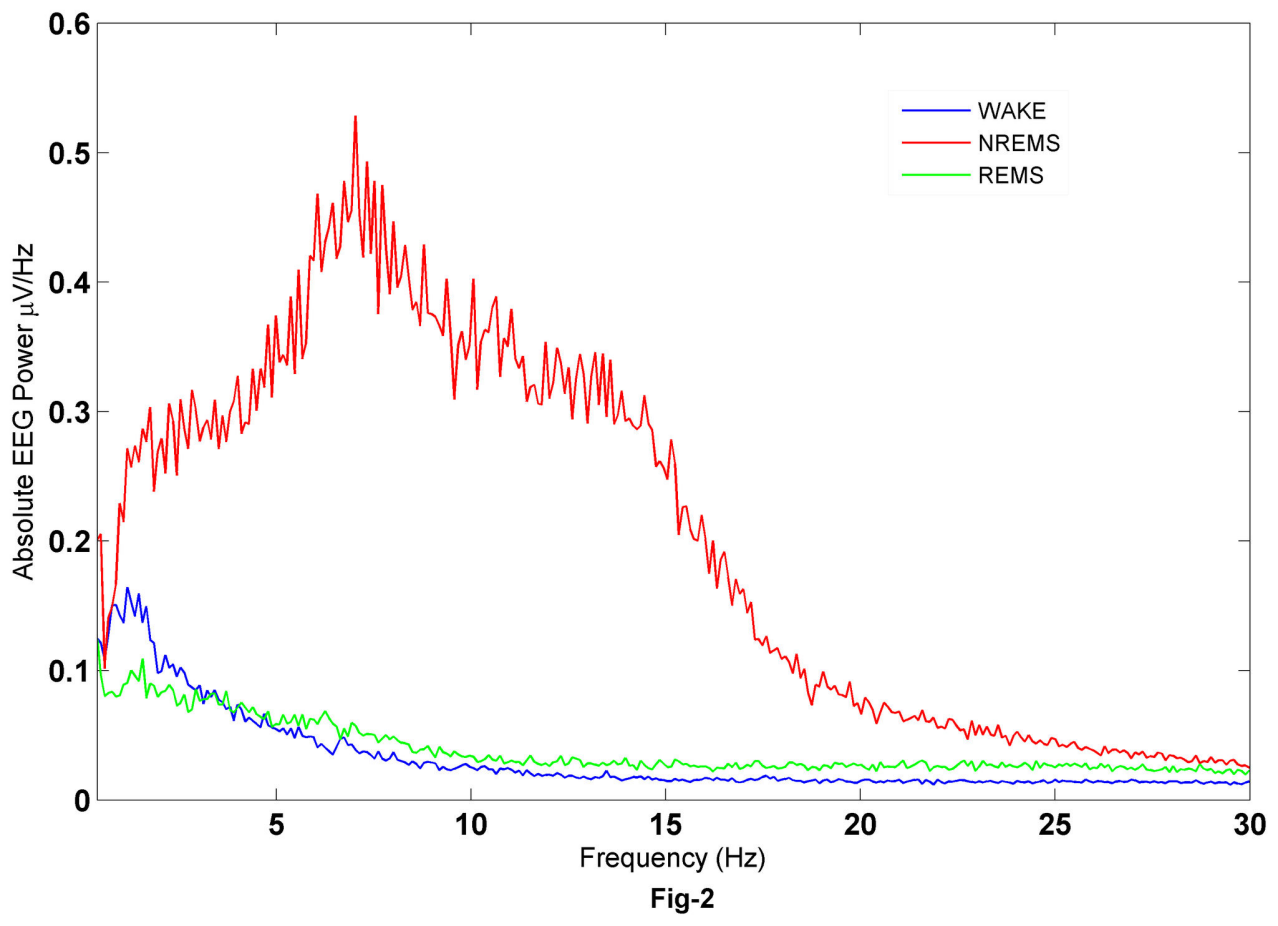
Fronto-frontal absolute EEG power spectral density (V2/Hz) in wake, NREMS and REMS of 10 sec epochs from n=12 rats. EEG power densities during NREMS were significantly higher than in REMS and wake. See text for details.

To verify the spectral power in the EEG during different sleep-wake states we computed the FFT of each 10s epoch collected from the baseline recording in a total of 12 rats. Figure 2 shows the power spectrum of the EEG during the different vigilance states and as can be seen there are clear differences in the power densities across the sleep-wake stages. The absolute power density during NREMS (2.0-20.0Hz) is higher than that in the wake and REMS. The power densities in waking were higher than in REMS from 0.5-4.0 Hz and lower from 4.0-30 Hz (Figure 2); this is consistent with earlier studies [14].

### Empirical Mode Decomposition

**Figure 3:**
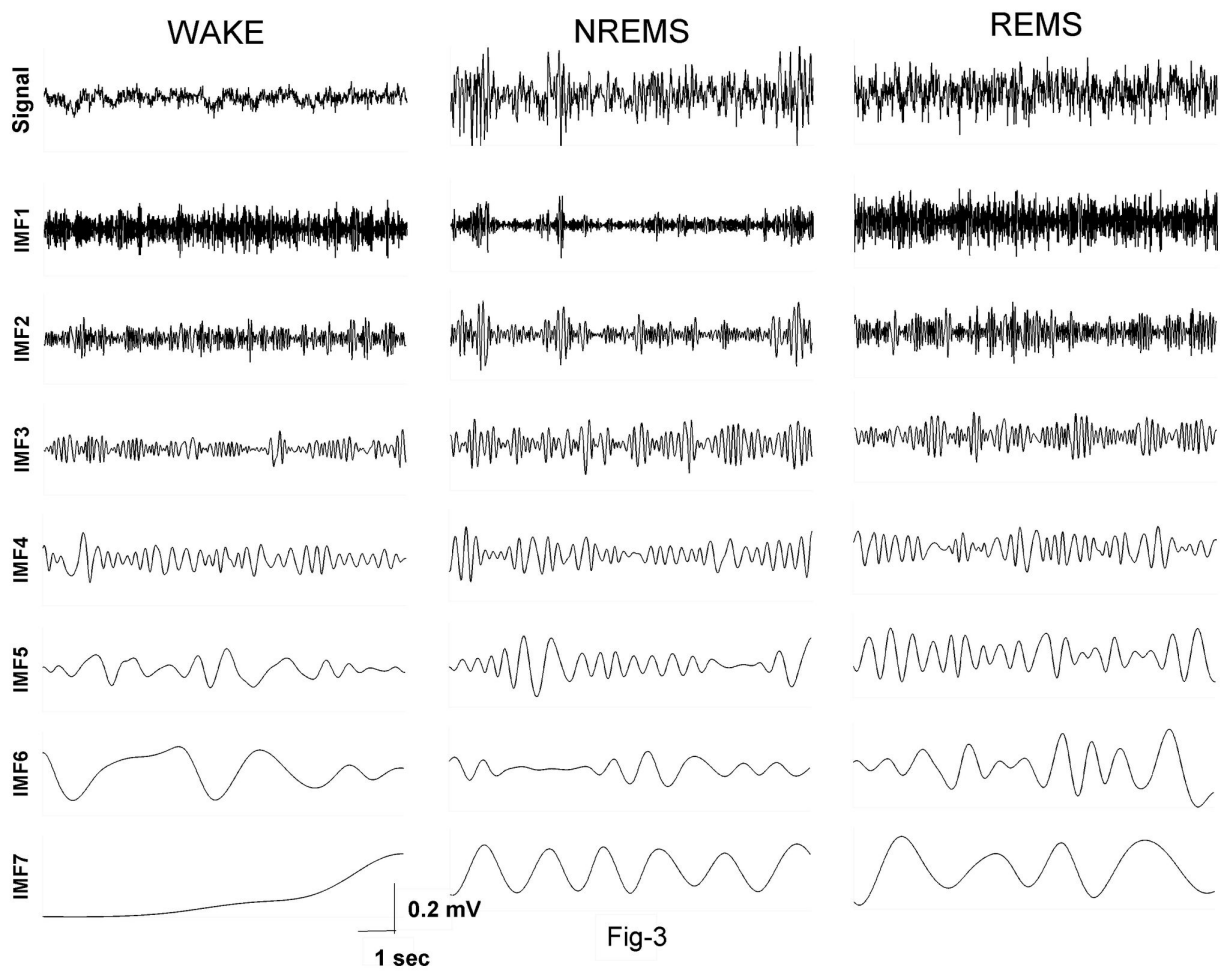
Empirical mode decomposition of Wake, NREMS and REMS. The first 7 IMFs are shown. The frequency gradually decreases as one move to lower IMFs.

**Figure 4:**
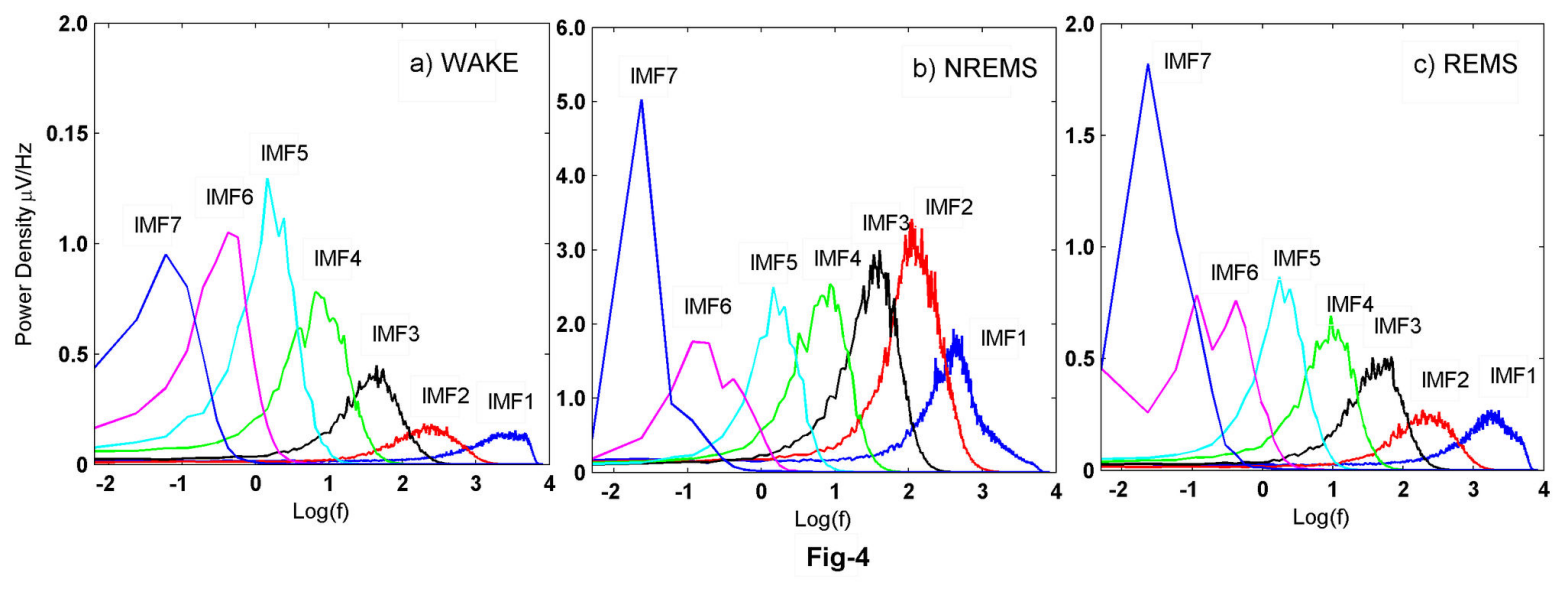
Power spectral density of IMFs in wake, NREMS and REMS. The PSD of NREMS is larger for all of the modes than wake and REMS. In IMF2 and IMF7, the PSD is different for wake and REMS. These particular IMFs can be considered as characteristic of vigilance state that they represent.

**Figure 5:**
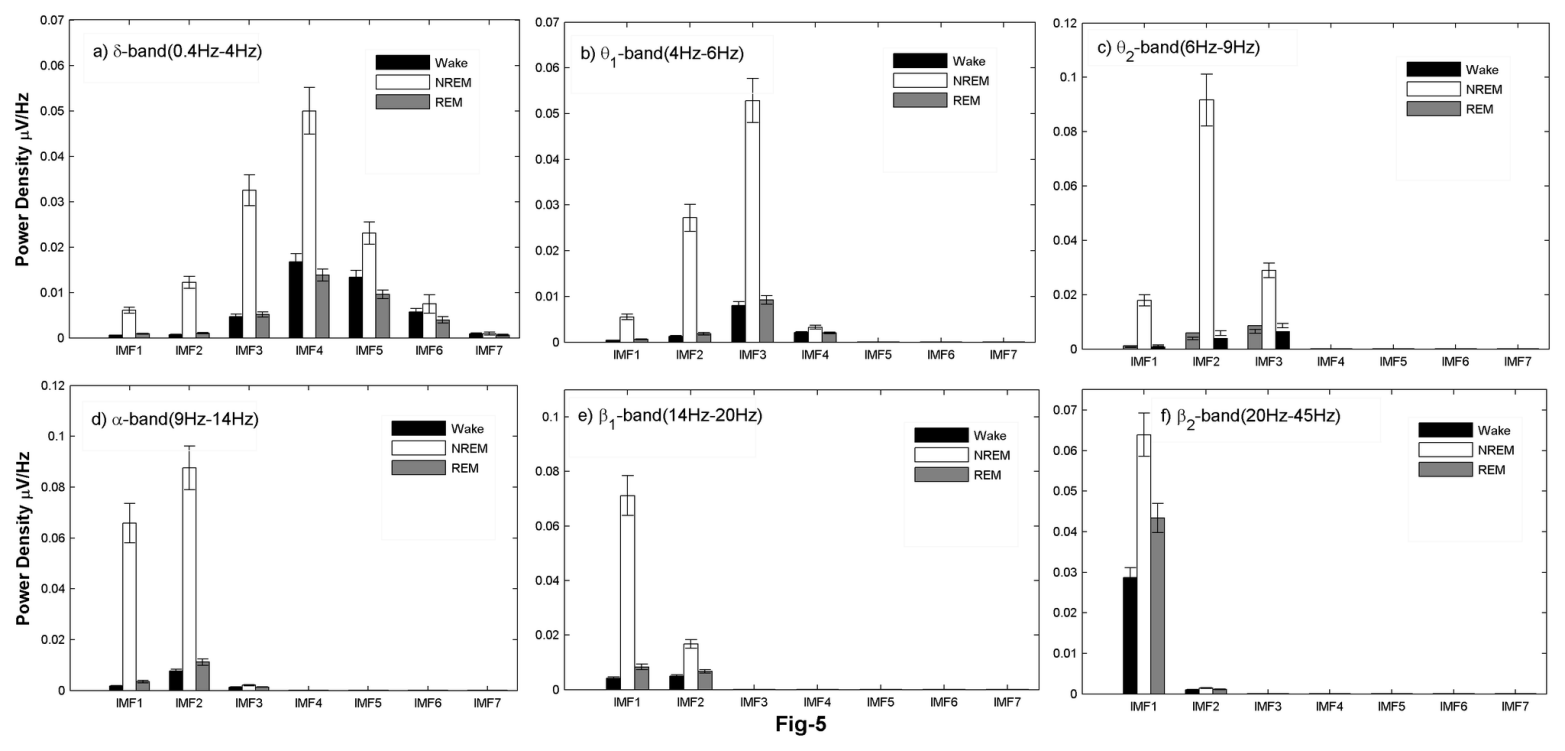
Average absolute power densities of 7 IMFs in different frequency bands. a) δ-band (0.4Hz-4Hz), b) θ_1_-band (4Hz-6Hz), c) θ_2_-band (6Hz-9Hz), d) α-band (9Hz-14Hz), e) β1-band (14Hz-20Hz) and f) β2-band (20Hz-45Hz). The PSD of NREMS is significantly more in all bands (for p-values see Table 1).

Each of the EEG epochs of 10 sec duration during different sleep-wake states was subjected to EMD. A standard implementation of the EMD algorithm yields about 10 IMFs, though typically the first 7 are significant (see Figure 3) and are subjected to further analysis. The frequency content of the remaining IMFs falls below a physiological bandwidth (≤ 0.1Hz). Figure 4 shows the average power densities of the leading 7 IMFs, denoted by IMF1 through IMF7. Characteristic changes are observed in the PSDs of the vigilance states, with the average trend being that the power densities in NREMS are more than wake and REMS. This is also accompanied by higher power in NREMS as can be seen in Figure 2. IMF spectral densities for wake and REMS follow the same trend. Closer inspection reveals that the PSD of IMF2 in waking state is higher than in REMS. Similarly the PSD of IMF7 in REMS exceeds the corresponding quantity in the wake, suggesting that the second and seventh modes carry information that is different for wake and REMS even though the EEG appears similar. We further categorized the absolute power densities into six frequency bands; δ (0.4Hz-4Hz), θ_1_ (4Hz-6Hz), θ_2_ (6Hz-9Hz), α (9Hz-14Hz), β_1_ (14Hz-20Hz) and β_2_ (20Hz-45Hz). As seen in Figure 5, the PSDs are distributed differently in different states. In all of the frequency bands, the PSDs in NREMS were the highest (Table 1).

**Table 1:** The PSD values of IMF's in δ (0.4-4Hz), θ1 (4-6Hz), θ2 (6-9Hz), α (9-14Hz), β1 (14-20Hz) and β2 (20-45Hz) bands.

Frequency Band	IMF	Wake	NREMS	REMS	F-value	p-value
δ-band(0.4Hz-4Hz)	IMF1	0.56±0.05	6.09±0.69	0.91±0.06	95.46	1.90E-14
	IMF2	0.71±0.10	12.25±1.26	1.01±0.12	129.19	2.22E-16
	IMF3	4.69±0.43	32.48±3.78	5.10±0.51	89.62	4.61E-14
	IMF4	16.77±1.38	49.99±5.52	13.82±1.05	73.48	6.99E-13
	IMF5	13.33±1.10	23.05±2.48	9.56±0.86	52.98	4.98E-11
θ1-band(4Hz-6Hz)	IMF1	0.37±0.03	5.51±0.54	0.61±0.04	139.60	1.11E-16
	IMF2	1.27±0.19	27.19±3.30	1.79±0.24	86.54	7.48E-14
	IMF3	7.99±0.89	52.82±5.06	9.24±0.82	154.25	0.00E+00
	IMF4	2.07±0.19	3.26±0.39	2.01±0.18		
θ2-band(6Hz-9Hz)	IMF1	0.69±0.07	17.86±2.05	1.25±0.12	107.95	3.33E-15
	IMF2	3.84±0.52	91.67±10.29	5.87±0.82	111.26	2.11E-15
	IMF3	6.44±0.61	28.93±2.39	8.59±0.72	186.03	0.00E+00
α-band(9Hz-14Hz)	IMF1	1.77±0.25	65.81±8.31	3.40±0.36	88.01	5.92E-14
	IMF2	7.61±0.79	87.55±9.04	11.17±1.10	151.41	0.00E+00
β1-band(14Hz-20Hz)	IMF1	4.09±0.53	71.13±8.57	8.32±0.77	85.20	9.29E-14
	IMF2	5.01±0.42	16.73±0.17	6.64±0.54	54.18	3.76E-11
β2-band(20Hz-45Hz)	IMF1	28.70±8.28	63.87±18.43	43.36±12.51	33.21	1.25E-08

All values are in nV/Hz±S.E.M. Since the IMFs form a complete and orthogonal set, one-way ANOVA with state (Wake, NREMS and REMS) as a factor were performed. The corresponding F and p-values are also given.

**Figure 6:**
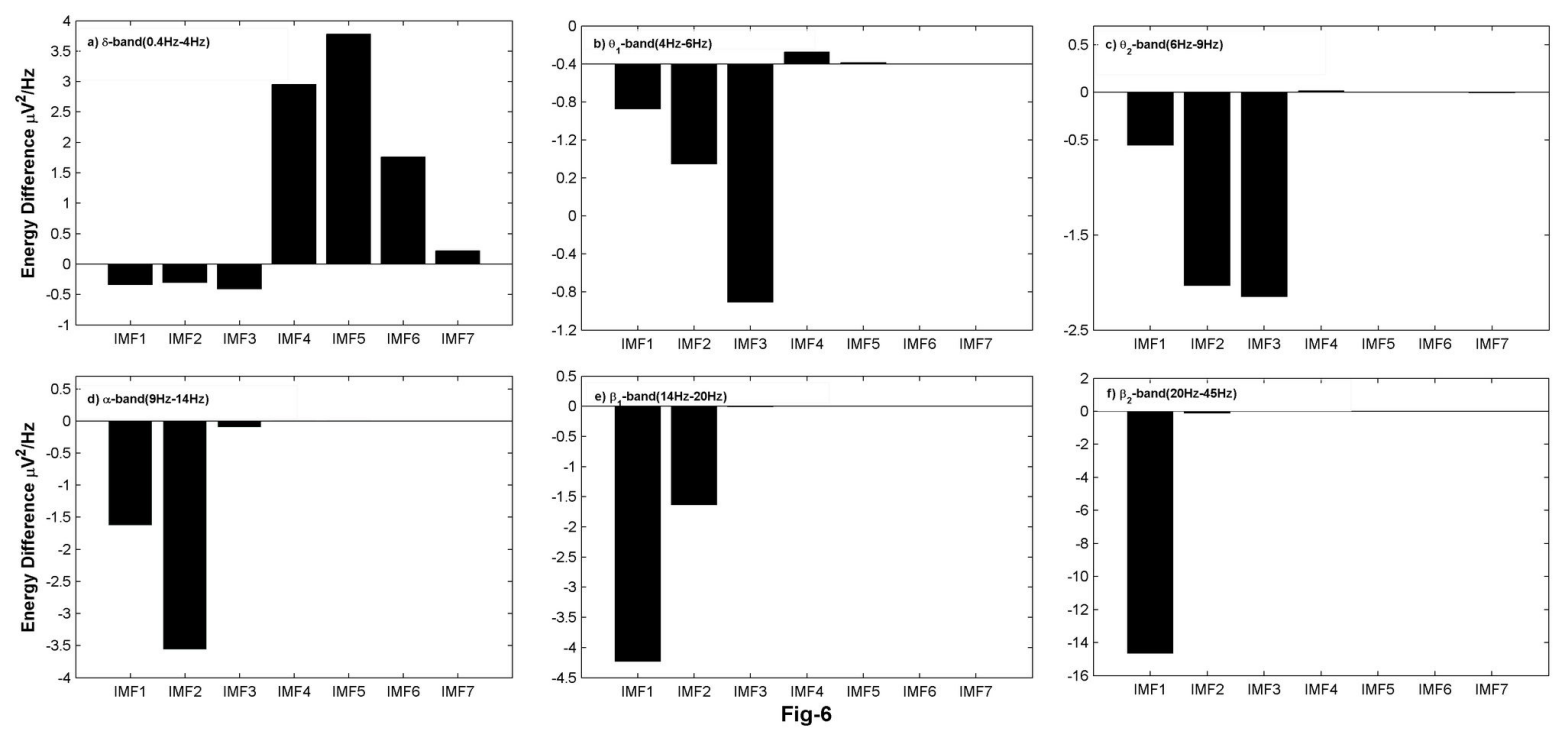
Changes in energy content of IMFs of wake relative to REMS state. PSD in δ-band for IMF4-IMF7 is higher during waking than REMS.

In order to quantify the energy content of the IMFs of the wake and REMS, the average difference in the energies for the two states are computed and are displayed in Figure 6. The PSD in the δ-band for IMF4-IMF6 are higher in wake state than in REMS. Unlike δ-band, all other bands show higher PSD for IMFs in REMS than in wake state. In higher frequency bands like β_1_ and β_2_, these values are negligible for IMF3-IMF7 (Figure 6e, f). However, the change in energy of the wake and REMS of IMF1 in β_2_ is larger, indicating that the oscillations occurring at this time scale are predominant in REMS. Thus, a detailed analysis of IMFs can provide insight into the basic dynamics that is characteristic of the individual states.

Another important measure that can be used to characterize the oscillations using EMD is the instantaneous phase. The IMFs obtained through EMD are symmetric with respect to the local zero mean and have the same number of zero-crossings and extrema [31] and therefore, the Hilbert transform gives meaningful instantaneous frequencies. The Hilbert transform properties of the IMFs in different sleep-wake states reveal the frequencies involved in the original signal at different time scales.

**Figure 7:**
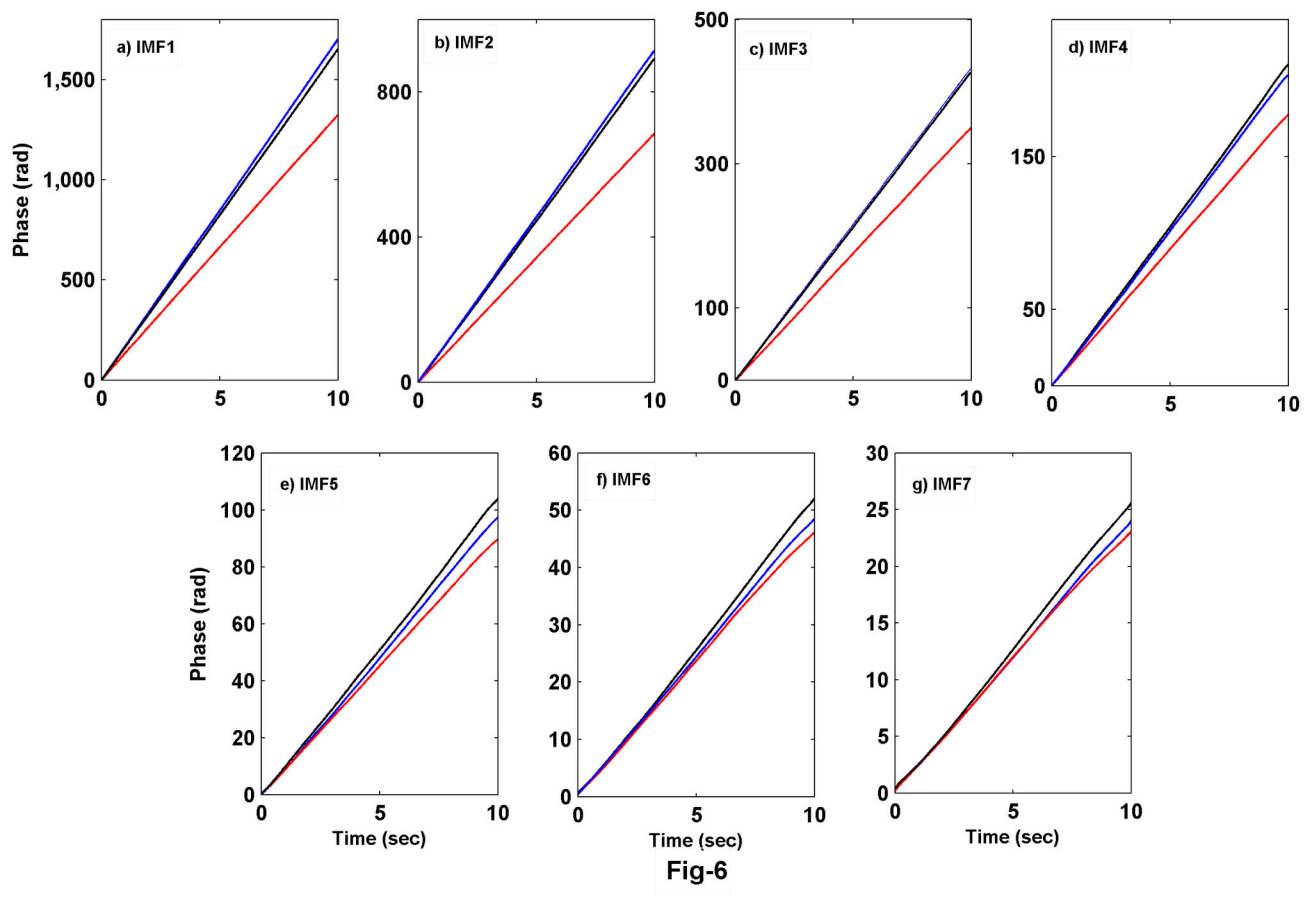
Unwrapped phase of the first 7 IMFs for three vigilance states are shown. The ranking of the rate of change of phase for wake and REMS is interchanged after the third IMF (Color code – Blue: wake, Red: NREMS and Black: REMS).

**Figure 8:**
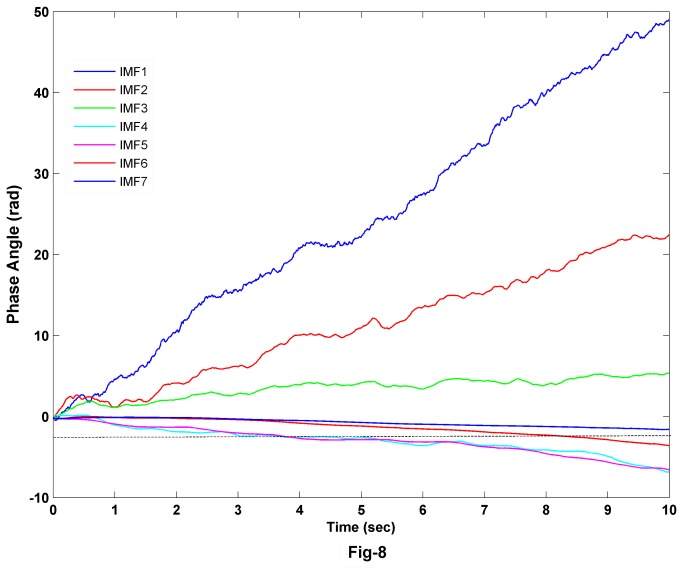
The change of phase of REMS relative to wake state for the 7 IMFs. The change of phase changes sign from negative to positive after the third IMF.

We calculated the instantaneous phase of each of the IMFs using Hilbert transform and these are shown in Figure 7. The slope of the phase function decreases from higher IMFs to lower IMFs (Note the scale of the y-axis in Figure 7 a-g). Similarly, the decrease in the phase function of the same IMF from wake to REMS to NREMS indicates the decrease of the mean frequency in these states respectively. The mean frequency for wake state is greater than in the REMS state in first two IMFs. However, this trend changes for IMF5-IMF7, in which the mean frequency of REMS state is more than wake state. To examine the changes in detail, we take the difference of phases for wake and REMS states as shown in Figure 8. The phase difference changes sign for IMF5-IMF7. These results suggest that the rate of information processing in waking state is more in the time scale described by IMF1-IMF3 than in REMS state. However, at the time scale described by IMF5-IMF7, the rate is more for REMS than that for wake. This result makes a clear distinction of local time scales involved across the wake and REMS EEG dynamics.

### Hilbert-Huang Spectral Entropy

**Figure 9:**
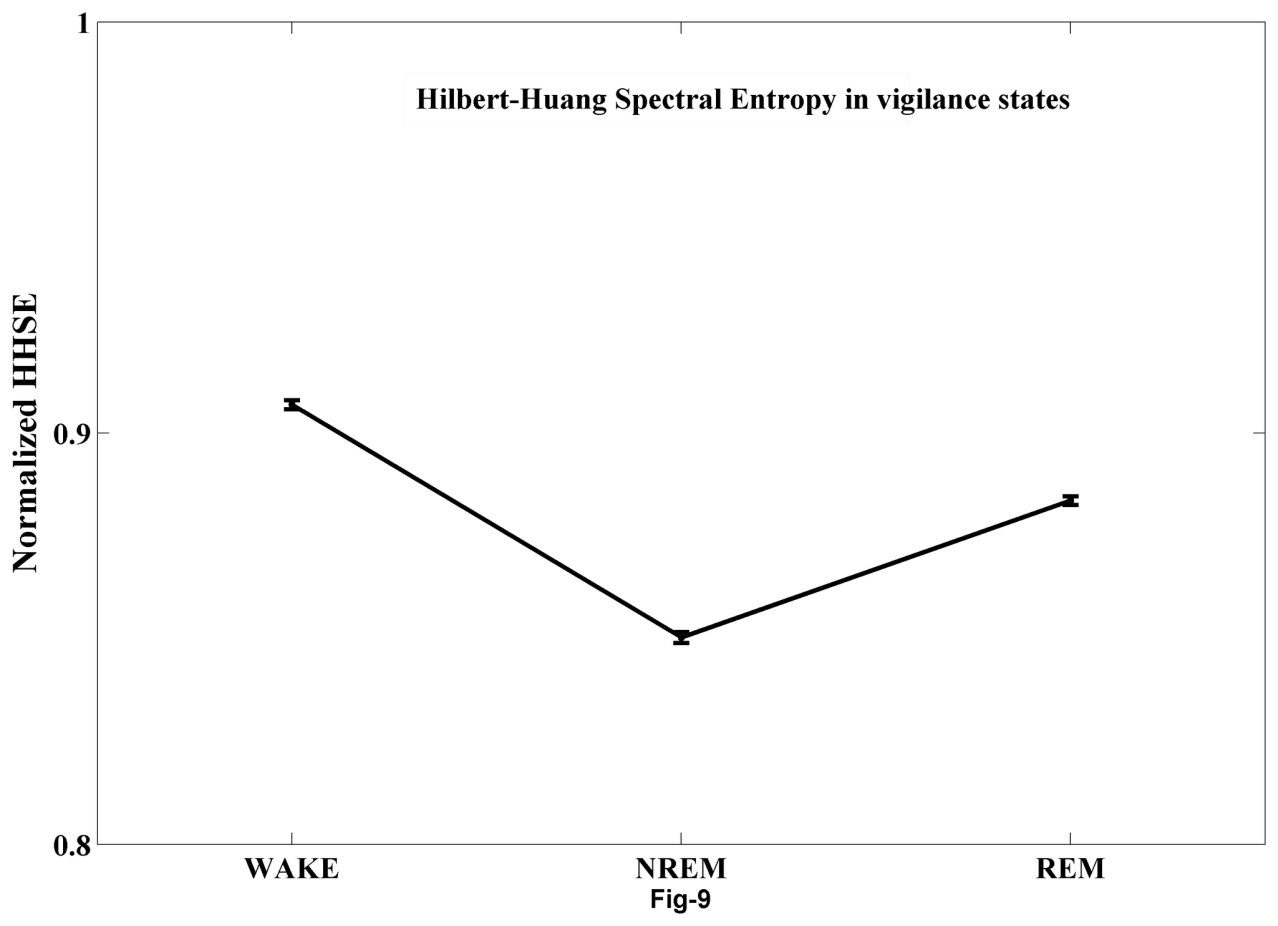
Hilbert–Huang spectral entropy for three vigilance states. HHSE during REMS is intermediate between wake and NREMS (n=12, F=148.22, p<0.01) and is significantly different than that of wake state (n=12, P<0.05).

Using information theory, the amount of information processed by each IMFs is characterized using Shannon entropy measures [36,37]. As previously mentioned in the methods section, marginal Hilbert spectrum is calculated for each of the EEG epochs in all of the three vigilance states and normalized HHSE is estimated using Eq. (9). The mean HHSE for wake, NREMS and REMS states are shown in Figure 9. Oneway ANOVA demonstrate a significant relation between HHSE and sleep wake states (F=148.22, p<0.001) and post-hoc pair wise comparison (Tukey test) demonstrated that the HHSE for wake is significantly higher than REMS (p<0.05). 

## Discussion

In the past few decades, sleep research has focused on identifying the brain structures involved in the regulation of sleep-wake states, the various neurotransmitters that play a role, and their mutual interactions; however, most of these are invasive studies. EEG serves as a hallmark of these neuro-anatomical and neuro-chemical mechanisms and it is therefore, important to understand the relevant aspects of EEG [38,39]. Our approach in the present study has been to decipher the local properties of the EEG in each of the sleep-wake states through a combination of nonlinear EMD and PSD based measures.

We find that the absolute PSD during the wake, NREMS and REMS is consistent with the findings of the earlier studies [14]. From Figure 2, it is evident that the PSD of NREMS is higher and span of bandwidth is more than other two states. This is evident even from visual observation: the NREMS EEG epochs contain high spindle density. This is usually considered as transition sleep as mentioned earlier under sleep scoring section. The peak in Figure 2 may be attributed to the burst discharge patterns (7Hz-14Hz) of thalamic reticular neurons involved in the generation of spindles [40].

Many spectral methods [13,41,42] and nonlinear dynamics tools [25,27,28] have been used to analyze EEG for discriminating various sleep-wake states. Power spectral analysis using FFT and linear models is useful for predicting the peak frequency and bandwidth of different vigilance states [43,44]. These methods are of limited applicability for EEG signals due to their intrinsic nonlinear and non-stationary structure. Non-linear measures such as the correlation dimension (CD), Lyapunov exponent (LE), Hurst exponent (HE) of sleep wakefulness all provide useful estimates regarding the composition of EEG in different vigilance states. The CD and LE decrease from light sleep to deep sleep and they increase during REMS [25,45]. However, these values are similar for wake and REMS and do not provide characteristic differences regarding these two states. The information gained from nonlinear measures is not redundant to that of obtained from spectral analysis.

We believe that an adaptive method such as EMD is more suitable for EEG analysis. The IMF power density displays variations across the states (Figs. 4, 5, 6). The PSD of the second and fifth IMFs give characteristics of the wake and REMS states, and provide useful insight into the frequencies involved in these states. Other studies using EMD have investigated diverse properties of EEG like detecting synchronization mediated by neuronal assemblies [46], analyzing the depth of anaesthesia [35] and detecting various phases (pre-ictal, seizure and ictal) of epilepsy [47]. Süleyman Baykut et al. [24] also employed EMD based techniques for discriminating sleep and wake states. Using EMD based energy ratios, they achieved faithful accuracy for discriminating vigilance states, but their method is not useful for discriminating the transition sleep, and since their method is based on energy ratios rather than energy bands, they do not shed light on frequency bands characteristic to each vigilance state which are important for locating and understanding sleep stage dependent generators.

In this study, we have analyzed the instantaneous phase and power content of each of the IMFs, obtained through EMD in different states. The rate of change of instantaneous phase decreases from wake to REMS to NREMS in the first few IMFs (e.g. IMF1-MF3). However, this trend changed to REMS to wake to NREMS for subsequent IMFs (IMF4-IMF7). This suggests that the IMFs show the characteristic oscillations at different time-scales involved in that particular state of EEG. We quantified the amount of information processing using the HHSE which has been used in estimating the depth of anesthesia [35]. The EEG shows apparent desynchronization during waking and REMS; however, these states are difficult to be classified using EEG characteristic alone. Our findings suggest that instantaneous phase of IMF1-IMF3 and HHSE decrease during REMS compared to that during waking. It is known that specific neurons containing various neurotransmitters are responsible for inducing changes in the EEG in relation to waking, NREMS and REMS. Whether these characteristic changes in the IMFs may be attributed to the specific changes in the levels of neurotransmitters and/or to specific neuronal activities related to waking, NREMS and REMS, require further investigation. Also, as we know that the neurons of the ascending reticular activating system are more active during waking, subject to verification, we propose that the changes in the physical properties of the EEG waves may be used as a non-invasive method to understand differential activities of neurons and their projections to the cortex. HHSE during waking is larger than that in REMS; it can thus provide a better estimate for the amount of information processed in these states.

Since HHSE is a measure of disorder in the system, we may say that during EEG desynchronization (waking or REMS) the brain is more temporally disordered than during NREMS state. However, since HHSE is higher during waking than during REMS, the former is more disordered state than the latter. Thus, our findings suggest that from waking to NREMS to REMS, the brain moves from (relatively) higher disorder to lower disorder and then to a state of intermediate disorder. The least disordered state is NREMS, and this separates the other two states whose disorder levels are higher; this supports our earlier proposed model [48]. Under normal conditions the transition of waking to REMS appears to be routed through a state of higher order, the NREMS. This also supports our proposition that all these states are playing on a background basal state, the “T” state [48]. These empirical results suggest that in addition to conventional spectral measures, the nonlinear EMD based measures provide useful information about the dynamics of the EEG during each of the vigilance states and may be suitable for healthcare applications for monitoring waking, NREMS and REMS. The differences of these local time quantitative measures at different locations, namely a topographic mapping, during different sleep-wake states can provide useful information for characterizing these states and their dependent generators.

## Conclusions

Our results indicate that dynamics of EEG during sleep-NREMS-REMS can be deciphered using EMD and spectral based methods. PSD analysis of EEG alone is not sufficient to distinguish wake and REMS states. PSD of IMF2 and IMF7 are characteristic of REMS and wake states, respectively. This indicates that the oscillations occurring at local time scales of IMF2 and IMF7 process information differently during these corresponding states. Similarly, the rate of change of phase increase for REMS as we move toward slow oscillations as indicated by higher IMFs. On a global scale, REMS is intermediately organized state between wake and NREMS states as is evident from the HHSE. These measures when experimented with neuro-chemical control of know brain areas involved in the regulation of each of wake-NREMS-REMS states can provide fruitful information regarding nature of neuronal oscillations involved in these states. Moreover, these measures can be useful to understand patho-physiological correlates of sleep-waking behavior.
